# Nudging children toward healthier food choices: An experiment combining school and home gardens

**DOI:** 10.1016/j.gfs.2020.100454

**Published:** 2020-09

**Authors:** Pepijn Schreinemachers, Ghassan Baliki, Rachana Manandhar Shrestha, Dhruba Raj Bhattarai, Ishwori P. Gautam, Puspa Lal Ghimire, Bhishma P. Subedi, Tilman Brück

**Affiliations:** a World Vegetable Center, Bangkok, Thailand; b Leibniz Institute of Vegetable and Ornamental Crops (IGZ), Großbeeren, Germany; c Department of Community and Global Health, Graduate School of Medicine, The University of Tokyo, Japan; d Outreach Research Division, Nepal Agricultural Research Council, Khumaltar, Lalitpur, Nepal; e National Horticulture Research Centre, Nepal Agricultural Research Council, Khumaltar, Lalitpur, Nepal; f Asia Network for Sustainable Agriculture and Bioresources (ANSAB), Baneshwor, Kathmandu, Nepal; g Natural Resources Institute (NRI), University of Greenwich, Chatham Maritime, UK; h ISDC – International Security and Development Center, Berlin, Germany

**Keywords:** Healthy eating, Impact evaluation, Nepal, Nutrition-sensitive agriculture, Randomized control trial, School garden

## Abstract

School gardens have become a widely used approach to influence children's food knowledge, preferences and choices in low- and high-income countries alike. However, evidence indicates that such programs are more effective at influencing food knowledge and preferences than actual food choices. Such finding may occur because school gardens insufficiently influence the food behavior of parents and because healthy food items are not always available in children's homes. We tested this hypothesis using a one-year cluster randomized controlled trial in Nepal with 15 treatment and 15 control schools and a matched sample of 779 schoolchildren (aged 8–12) and their caregivers. Data were collected before and after the intervention during the 2018–2019 school year. In addition, children's food consumption was monitored using a monthly food logbook. Average treatment effects were quantified with a double-difference estimator. For caregivers, the intervention led to a 26% increase in their food and nutrition knowledge (p < 0.001), a 5% increase in their agricultural knowledge (p = 0.022), a 10% increase in their liking for vegetables (p < 0.001), and a 15% increase in home garden productivity (p = 0.073). For children, the intervention had no discernible effect on food and nutrition knowledge (p = 0.666) but led to a 6% increase in their liking for vegetables (p = 0.070), healthy food practices (p < 0.001), and vegetable consumption (October–December +15%; p = 0.084; January–March +26%; p = 0.017; April–June +26%; p = 0.088). The results therefore indicate both schools and parents matter for nudging children toward healthier food choices.

## Highlights

• Past studies showed limited effects of school gardens on children's food choices.
• We test a novel design that trains children and caregivers in gardening and nutrition.
• The cluster-RCT included 30 schools and 779 children/caregivers in Nepal.
• Children's rate of vegetable consumption increased 15–26%, depending on season.
• School gardens need to affect household-level food availability/preferences.

## 1 Introduction

Evidence indicates that it is critically important to develop healthy food preferences and eating habits in children because these can persist into adolescence and adulthood (Birch et al., 2007; Cooke, 2007; Kelder et al., 1994; Wadhera et al., 2015). Many interventions therefore aim to nudge children toward healthier eating habits to obtain long-term and even lifetime improvements in nutrition and health. School garden programs are one such intervention trying to instill healthier eating habits in children, and are increasingly common in high- and low-income countries alike (Benkowitz et al., 2019; Christian et al., 2014; FAO, 2005; Hunter et al., 2020; Hutchinson et al., 2015; Huys et al., 2019; Nury et al., 2017; Ozer, 2007; Parmer et al., 2009; Triador et al., 2015). Through a combination of hands-on experience with gardening and nutritional education, children learn how to grow, appreciate and like healthy foods such as fruit and vegetables, which tend to be under-consumed.

Compelling as the concept may be, evidence for the nutritional impact of school gardens remains limited. The current evidence basis largely relies on studies for high-income countries. A review of 12 quantitative studies in the United States found positive outcomes in the area of science achievement (knowledge) for 9 schools but increased fruit and vegetable consumption for only 1 school (Blair, 2009). Another review of studies for Australia, the United States and Europe found significant effects on healthier food preferences in 8 out of 13 studies, improvements in food knowledge and attitudes in 7 out of 10 studies, but a significant increase in children's fruit and vegetable consumption in only 2 of the 13 studies (Ohly et al., 2016). More recently, an evaluation of a school garden program in Belgium found small but significant effects on knowledge and awareness, but no significant effect on vegetable consumption (Huys et al., 2019). These studies therefore show that school garden programs tend to be more effective in improving children's knowledge, attitudes and preferences than at changing actual food behavior.

Three randomized controlled trials conducted in low-income countries broadly confirm these observations (summarized in Schreinemachers et al., 2020). All studies come from our own research group. To our knowledge, there are no other rigorous studies that have evaluated school garden programs in low-income countries. The study for Nepal showed a positive effect on children's awareness of vegetables, their knowledge of agriculture and of food and nutrition, and their stated preferences for vegetables, but no significant effect on fruit or vegetable consumption (Schreinemachers et al., 2017a). For Burkina Faso, there were no significant effects except for food and nutrition knowledge (Schreinemachers et al., 2019). For Bhutan, there were positive effects on awareness, knowledge, and preferences and an increase in the probability of children consuming vegetables, with a positive association between vegetable consumption and children having a vegetable garden at home (Schreinemachers et al., 2017b).

A review of factors influencing children's food behavior (Scaglioni et al., 2018) identified parental food habits as the most important factor. Personal habits provide a possible explanation for the weak effect of school gardens on children's food behavior: children eat most of their meals at home rather than at school and parents (often mothers) generally decide what meals are served. Another factor limiting children's food choice may be that healthy food items such as fruit and vegetables are not always available at home, especially in poor rural households or year-round. Low availability may explain why the Bhutan study found a positive association between home gardens and children's vegetable consumption. Other studies also demonstrated that home garden interventions can increase household vegetable production and consumption in the South Asian context (Baliki et al., 2019; Bird et al., 2019; Osei et al., 2016; Schreinemachers et al., 2016).

These two explanations lead to the hypothesis that school gardens can nudge children toward healthier food choices if such programs simultaneously influence the food behavior of parents and increase the availability of healthy food items within the household. The study tests this hypothesis with data from a randomized controlled trial of a novel school garden project in Nepal that supported 15 (out of a total sample of 30) schools to implement school gardens and provided home garden training and nutrition education to the children's caregivers.

The hypothesis is important to improve the design of school garden programs as evidence to date, reviewed above, shows only weak impact on children's fruit and vegetable intake. Several studies have pointed at the importance of multi-component school-based interventions. For instance, a structured literature review of school-based interventions concluded that combinations of classroom curriculum, parent and food service components show the greatest promise for increased fruit and vegetable consumption among children (Blanchette and Brug, 2006). In addition, reviews by Rasmussen et al. (2006) and Scaglioni et al. (2018) showed that increasing children's access to fruit and vegetables at home and greater parental intake are both associated with increased fruit and vegetable consumption among children. Our study also contributes to deepening our understanding of factors driving healthy food choices among children in low-income countries, which is important in the context of dietary trends toward increased consumption of highly-processed foods and beverages and rising prevalence of overweight and obesity among children and adolescents (Abarca-Gómez et al., 2017; Ng et al., 2014; Popkin et al., 2012).

## 2 Methods and data

### 2.1 Choice of study location

Nepal was selected for the study in order to build on a previous school garden project that had designed and tested a school garden model (Schreinemachers et al., 2017a; Shrestha et al., 2020). Furthermore, the government of Nepal has showed much interest in school gardens as it fits the country's Multi-sector Nutrition Plan, which emphasizes the need for combining health, education, agriculture and social welfare for addressing malnutrition in the country (Government of Nepal, 2017). Nepal has made good progress reducing malnutrition (Headey and Hoddinott, 2015), but stunting continues to affect 32% of adolescent boys and girls and anemia prevalence is 21% for adolescent girls (Ministry of Health and Population et al., 2012). Unbalanced diets are identified as one of the causes of malnutrition. For instance, it has been reported that children under the age of two in Kathmandu Valley are getting a quarter of their calories from unhealthy snack foods and beverages (Pries et al., 2019), which is indicative of a wider problem of unhealthy eating habits. Another study reported that only 1.1% of Nepal's adult population consumes 400 g of fruits and vegetables a day—the amount recommended by the World Health Organization (Frank et al., 2019).

Within Nepal, the study was conducted in Sindhupalchok District, located between Kathmandu and the border with China. The district's area is 2542 km^2^ and had about 290,000 residents in 2011, the year of the last census (CBS, 2018). About 25% of the district population lived below the national poverty line in 2011, which was about the same as the national average (World Bank, 2011). It has extreme altitude differences, ranging from 850 to 7000 m above sea level. The district was severely affected by the Nepal Ghorka Earthquake of April 25, 2015.

### 2.2 Program theory and intervention design

The program theory posits that hands-on gardening experience and complementary lessons at school strengthen children's knowledge about the importance of good nutrition. As a result, children are expected to develop a more positive attitude toward eating vegetables (and healthy eating more generally). These changes would be reinforced and supported at home as parents gain better skills in gardening and feel motivated to grow vegetables after receiving seed packs, garden training and a better understanding of how vegetables contribute to family health. It is not expected that school gardens supply substantial quantities of vegetables; the purpose of a school garden is as an educational tool while home gardens or local markets would be the main source of increased vegetable supplies. A stronger interest of children and parents in vegetables combined with their increased availability and accessibility is expected to have a positive effect on children's vegetable consumption.

The school garden intervention consisted of a physical garden for hands-on experience in vegetable growing and nutrition education following a booklet with 23 weekly learning modules (Bhattarai et al., 2016). It involved children in grades 4 and 5 (aged 8–12 years old). We selected these early grades assuming that the food behavior of young children can be influenced more easily, while they are old enough to do physical work in the garden and do the nutrition learning modules. Two teachers per school were trained in running the school garden, of whom one was designated as school garden focal teacher and was responsible for the implementation. Schools were given three periodic cash installments of (US$ 440, 220 and 220). The money had to be spent on land preparation, a water tank, garden tools, plastic sheets for making a nursery and fencing materials as specified in contracts signed between each school and the implementing agency. The garden was usually established on the school ground, but a few schools needed to rent land. The average garden was 90 m^2^ in size (the range was 32–240 m^2^). Seed of nine local vegetable varieties was distributed for the winter season (cauliflower, radish, carrot, pea, broad leaf mustard, turnip, broccoli, fenugreek, spinach) and seed of another ten varieties was distributed for the summer season (soybean, swiss chard, capsicum, coriander, bitter gourd, eggplant, okra, pumpkin, yard long bean, tomato). Each school received at least two technical support visits by a trained staff.

As part of the intervention, children's caregivers additionally received support to improve their home gardens. The term “caregivers” here refers to the main person in the household taking care of a child. It is usually the mother, but sometimes it is the father or grandmother. In some households, for example, parents were working in Kathmandu or abroad and the grandmother was the caregiver. The home garden training consisted of three periodic sessions on gardening and nutrition. The training used a bi-modular agricultural and nutrition manual developed specifically for the project. The garden-based training included topics such as garden establishment, crop rotation, compost making, pest management and seed saving. Nutrition training included topics such as the role of vegetables for family health, the nutritional content of different food items, and cooking methods to preserve the nutritional quality of vegetables. About 80% of the caregivers participated in the nutrition training. In addition to the training, each caregiver received 155 g of seed of 9 different vegetables for the winter season and again 116 g of 10 different vegetables for the summer season. Caregivers and schools were supplied with the same varieties. Caregivers also received Effective Microorganism (EM) during the winter season for preparing quality compost and biopesticides to deal with red ants and aphids for the summer season (as project staff noticed that these were a key problem). School garden focal teachers provided technical backstopping to the caregivers and visited their home gardens on Saturdays. The teachers were paid by the project to do this. The visits of school teachers to parents' home to observe their garden was expected to create an additional nudge to motivate children and caregivers to implement their garden well.

The Nepal Agricultural Research Council (NARC) implemented the school-garden component while the Asia Network for Sustainable Agriculture and Bioresources (ANSAB) implemented the home garden component. Senior project staff of these organizations conducted two monitoring visits to oversee progress in the project implementation. The research team conducted one monitoring visit in December 2018, including focus group discussions with teachers, parents and children in five treatment schools to learn about the implementation process and challenges encountered.

### 2.3 Outcome variables

The primary outcome variable is the proportion of children's meals that included vegetables recorded using a 24-h recall method. The data were self-reported by the children using food logbooks. Children were asked to list all food items they ate or drank for breakfast, lunch, afternoon snack, evening snack, and dinner—as based on the common meal pattern in Nepal. During the data entry, each meal was coded as 0 (no vegetable consumed) or 1 (some vegetable consumed). This information was used to calculate the proportion of meals that included vegetables with the denominator being the total number of meals a child had consumed on a particular day. These data were recorded for the baseline and endline surveys and for every month in between. The baseline and endline were monitored by enumerators while school teachers monitored the data entry for the other months.

Secondary outcome variables were selected along the pathway from knowledge creation to behavior change. These can be considered as intermediary outcomes and help to understand the critical stages in the program's theory of change.

*Food and nutrition knowledge* were measured using 15 multiple choice questions with four answer options each of which exactly one was factually correct. The questions probed about the association between food and body functions (e.g. “Which food is good for your eyes? 1. Cucumber; 2. Beans; 3. Carrots; 4. Chicken meat”), about nutrients lodged in food (e.g. “Which food has lots of Vitamin C? 1. Carrots; 2. Chicken meat; 3. Lemons; 4. Rice”), and about healthy diets (e.g. “Which food is not part of a healthy diet? 1. Vegetables; 2. Carbonated drinks; 3. Meat; 4. Fruit”). The variable was expressed as the proportion of correct answers and was recorded for children and caregivers separately.

*Agricultural knowledge* was measured using 14 photos of common garden pests (e.g. snail, caterpillar) and beneficial insects (e.g. bee, ladybug). Children and caregivers were asked to tick all photos of insects that are potentially harmful to plants. The variable was expressed as the proportion of correct answers, ranging from 0 to 1.

*Liking for vegetables* was measured by showing respondents 15 photos of vegetables and recording their liking as 4 (like it a lot), 3 (like it), 2 (neutral), 1 (don't like it). If the person didn't know the vegetable then the answer was recorded as 5 and excluded from the analysis. The mean liking for vegetables was calculated and brought into the range [0,1] by using unity-based normalization ([value-min]/[max-min]). Answers were recorded for children and caregivers separately. In addition, caregivers were similarly asked to record their perception of their children's liking of vegetables.

*Snack choices* were recorded for children through 10 questions. Each question presented photos of three common snack items, including one healthier item and two less healthy items. Children had to choose the item he or she liked most to eat as a snack. The variable was expressed as the proportion of healthier snack choices, ranging from 0 to 1.

*Food practices* were measured using 8 statements that were read out to the caregivers. Examples are “Children in my household buy junk food” and “Children in my household eat a meal before going to school”. Possible answers included: Never (1), Rarely (2), Often (3), and Very often (4). The mean liking for vegetables was calculated and brought into the range [0,1] by using unity-based normalization.

With regard to the home gardens, the interviews with caregivers recorded the names of different vegetables produced in different seasons, garden practices used in the home garden (e.g. compost making, raised planting beds), and challenges encountered in the home garden.

### 2.4 Study design and sample size

**Fig. 1:**
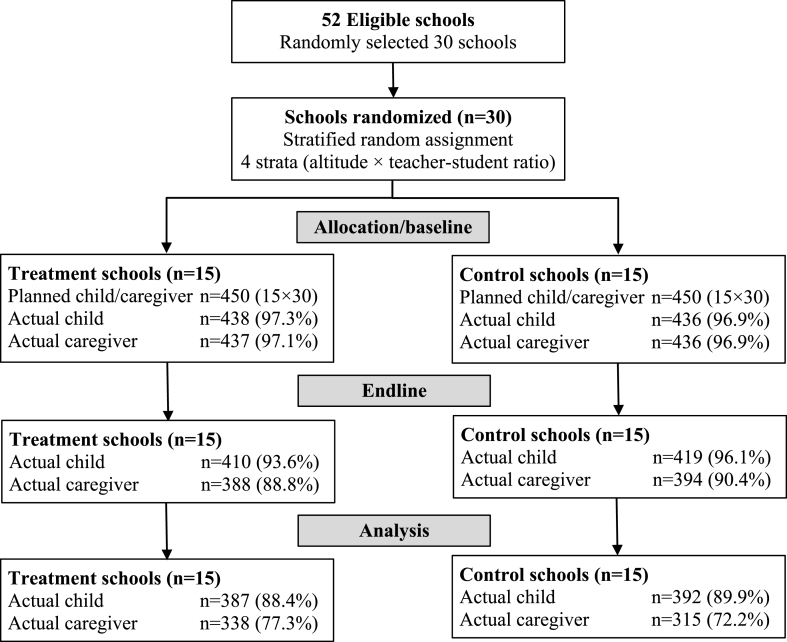
Consort flow diagram for the study.

The study used a cluster randomized controlled trial design in which villages (and their schools) were randomly assigned to either a control group or a treatment group. The treatment group received school gardens and complementary home gardens while the control group received neither intervention during the study period. The consort flow diagram in Fig. 1 describes the sample selection process.

Small sample size is a common challenge in the evaluation of school-based programs. The unit of intervention is the school and it is usually impractical to include many schools at the pilot stage. Previous evaluations of school garden programs in high-income countries used between one and five schools and collected data for no more than 500 children (Blair, 2009). One recent study for the UK used two treatment arms and a total sample of 23 schools and 1391 children (Christian et al., 2014). The previous study in Nepal is the most extensive study to date and included 30 schools and 1570 children (Schreinemachers et al., 2017a).

Power calculations were used to decide on the sampling strategy. We derived the minimum detectable difference (DD = 0.20) and intra-cluster correlation coefficient (ICC = 0.025) from the previous study on school gardens in Nepal (Schreinemachers et al., 2017a). These values were based on related outcome variables, including the share of children who ate fruit and vegetables, the number of fruits and vegetables consumed, and share of correct answers on knowledge tests for nutrition and sustainable agriculture. Holding the statistical power threshold constant at 0.8 and using a 95% confidence interval, we ran simulations varying the number of schools and sample of children per school. The simulations indicated that the study would be sufficiently powered using 30 schools and 30 matched children-households per school.

A problem with a lack of balance in outcome variables may arise given the small sample of schools (clusters). Two strategies were applied to deal with this. First, eligibility criteria were used to reduce the variation between clusters. We selected non-boarding government-run schools with access to a source of water for irrigation. The use of eligibility criteria increases the internal validity of the study by making schools more comparable, but there is a trade-off in external validity as the results cannot be generalized to all schools. Limiting the selection criteria is necessary and justified when experimenting with a novel intervention. Second, we used sample stratification to increase the likelihood of balance. Bruhn and McKenzie (2009) showed that stratification performs particularly well in small sample experiments. Altitude (as a proxy of the agroclimatic conditions) and the teacher-student ratio (as a proxy of school quality) were used as stratification variables to ensure balance between the two groups.

Schools were selected from six rural municipalities (*palikas*) of Sindhupalchok District that were relatively easy to access (Chautra, Indrawati, Melamchi, Sunkoshi, Lisankhu Pakhar, Barabisha). A list of 52 schools that met the eligibility criteria in these locations was created. The list was completed together with the local district education office. These 52 schools represent about 10% of all primary schools in the district, but may not be representative for all schools. Thirty schools were randomly selected from this list for inclusion in the study. Secondary data were collected on the above-mentioned stratification variables. From each stratum, we randomly assigned half of the schools to the treatment and the other half to the control.

Project participation of children and caregivers in the treatment group was complete, but not all caregivers participated in every training event. Sample attrition between baseline and endline was 5.1% for the sample of children, but for the sample of caregivers it was 11.7% for the treatment and 10.2% for the control. A comparison between attrited and non-attrited households showed no significant differences in means (p < 0.05), which suggests that sample attrition is not a source of bias in this study.

### 2.5 Research ethics

The study was approved by the Nepal Health Research Council (NHRC) Ethical Review Board on May 30, 2018 (Reg. No. 222/2018). It was also approved by the Institutional Biosafety and Research Ethics Board of the World Vegetable Center (Approval No. 23). Study participation was voluntary for all children and caregivers. School principals and caregivers signed a written consent form for themselves and for their children. Participation in the project bore no risk for parents and children while the potential benefits in terms of improved nutrition as a result of the school and home garden intervention were potentially substantial. The project supported the control schools to establish a school garden after the completion of the endline data collection, which was an important incentive for control schools to participate in the project. The trial is included in the Registry for International Development Impact Evaluations (RIDIE; Study ID 5cd93ec673096).

### 2.6 Data collection and analysis

The study administered a baseline survey at the start of the school year in June 2018 and an endline survey in June 2019. The surveys were done in the same month to control for seasonal variations in the supply of fresh food. Data were collected from the schoolchildren and their respective caregivers. We randomly selected 15 children each from grades 4 and 5 of each school if there were more than 15 children in a grade. The data set is publicly available on Harvard Dataverse (Schreinemachers, 2020).

We quantified the average treatment effect (ATE), which is the change in outcomes for the treatment group minus the change in outcomes for the control group, using a difference-in-difference estimator. The method eliminates the effect of selection bias, if present. The key assumption is that the average change in the control group represents the counterfactual change in the treatment group in the absence of the project. The so-called “parallel paths” assumption is likely to hold because the treatment was randomly assigned and the intervention period is short (see also below for empirical evidence supporting this assumption). A cluster effect was added to all regression models because schools are the unit of intervention but children and households are the unit of observation. Means, standard deviations and t-values were also cluster-adjusted.

## 3 Results

The mean age of schoolchildren in the sample is 10 years and 55% are girls (Table 1). On average children walk about 25 min to school, though many children walk much longer as shown by the high standard deviation. For 81% of the children, the caregiver is their mother, but for 5% it is their grandmother, and for 5% it is their father. For the remaining children, the caregiver may be an aunt or older sister. Most of the caregivers are engaged in farming (74%). About 38% of the caregivers are able to read and write.

**Table 1:** Mean baseline characteristics for children, caregivers and households for control and treatment, 2018.

Characteristic	Control (n = 392)		Treatment (n = 387)		p-value^1^
	Mean	SD	Mean	SD	
Schoolchildren:					
Age (years)	10.43	1.55	10.33	1.49	0.677
Female (prop.)	0.56	0.50	0.55	0.50	0.836
Grade 4 (prop.)	0.48	0.50	0.51	0.50	0.523
Distance to school (minutes)	26.27	24.34	24.13	23.03	0.487
Caregivers:					
Age (years)	35.51	9.02	35.35	10.02	0.863
Female (prop.)	0.93	0.25	0.93	0.25	0.973
Mother (prop.)	0.83	0.37	0.79	0.41	0.290
Father (prop.)	0.05	0.22	0.06	0.25	0.500
Grandmother (prop.)	0.05	0.22	0.05	0.21	0.922
Able to read and write (prop.)	0.38	0.48	0.39	0.49	0.830
Main occupation farming (prop.)	0.74	0.44	0.74	0.44	0.976
Household:					
Household size (persons)	5.41	1.97	5.55	1.84	0.368
Includes a grandmother (prop.)	0.24	0.43	0.28	0.45	0.271
Sells vegetables (prop.)	0.15	0.36	0.18	0.38	0.503
Mother prepares meals (prop.)	0.84	0.37	0.81	0.39	0.461
Grandmother prepares meals (prop.)	0.06	0.24	0.07	0.26	0.585

Note: ^1^ Welch two sample *t*-test with unequal and clustered variance. Prop. = proportion.

A comparison of means for general characteristics of the children, caregivers and households included in the study indicates that the sample is balanced at baseline (Table 1). Most importantly, a comparison of mean outcomes at baseline does not show any differences significant at a 95% confidence interval (Table 2, Table 3, Table 4). This finding gives confidence that the stratified random assignment created a balanced sample.

**Table 2:** Baseline and endline means and average treatment effects on children's and caregivers' knowledge and preferences.

Outcome (proportions)	Baseline			Endline			Impact	
	C	T	p-value	C	T	p-value	ATE	p-value
Food and nutrition knowledge:								
Children	0.48	0.49	0.431	0.54	0.57	0.213	0.01	0.666
	(0.15)	(0.14)		(0.15)	(0.15)		(0.03)	
Caregivers	0.53	0.53	0.919	0.57	0.70	<0.001	0.14	<0.001
	(0.17)	(0.17)		(0.17)	(0.16)		(0.03)	
Agricultural knowledge:								
Children	0.52	0.53	0.466	0.53	0.57	0.003	0.03	0.119
	(0.11)	(0.12)		(0.12)	(0.12)		(0.02)	
Caregivers	0.59	0.59	0.654	0.58	0.61	<0.001	0.03	0.022
	(0.12)	(0.12)		(0.11)	(0.10)		(0.01)	
Liking for vegetables:								
Children	0.63	0.64	0.700	0.58	0.63	0.021	0.04	0.070
	(0.18)	(0.18)		(0.18)	(0.17)		(0.02)	
Caregivers	0.59	0.59	0.943	0.53	0.60	<0.001	0.06	<0.001
	(0.17)	(0.17)		(0.15)	(0.18)		(0.02)	
Caregivers' perception of children's liking	0.56	0.58	0.191	0.54	0.63	<0.001	0.06	<0.001
	(0.18)	(0.18)		(0.17)	(0.16)		(0.02)	
Children's healthy snack preferences	0.63	0.61	0.541	0.66	0.69	0.257	0.05	0.042
	(0.22)	(0.22)		(0.23)	(0.22)		(0.03)	

Notes: C=Control; T = Treatment. The numbers in parentheses indicate standard deviations for the means and standard errors for the average treatment effect (ATE).

**Table 3:** Baseline and endline means and average treatment effects for home garden management practices and vegetable production.

Outcome	Baseline			Endline			Impact	
	C	T	p-value	C	T	p-value	ATE	p-value
Technology adoption (prop. of households):								
Seed packs	0.95 (0.23)	0.85 (0.36)	<0.001	0.89 (0.32)	0.92 (0.28)	0.411	0.12 (0.04)	0.002
Own seed saving	0.50 (0.50)	0.43 (0.50)	0.138	0.48 (0.50)	0.54 (0.50)	0.238	0.13 (0.06)	0.033
Pruning	0.09 (0.29)	0.12 (0.33)	0.369	0.15 (0.35)	0.29 (0.45)	0.013	0.11 (0.07)	0.096
Sick plant removal	0.46 (0.50)	0.40 (0.49)	0.255	0.65 (0.48)	0.71 (0.46)	0.171	0.12 (0.07)	0.082
Compost making	0.22 (0.41)	0.24 (0.43)	0.588	0.22 (0.41)	0.25 (0.43)	0.434	0.01 (0.05)	0.835
Raised beds	0.35 (0.48)	0.36 (0.48)	0.838	0.54 (0.50)	0.63 (0.48)	0.134	0.07 (0.09)	0.425
Seedling nursery	0.28 (0.45)	0.30 (0.46)	0.664	0.26 (0.44)	0.35 (0.48)	0.147	0.06 (0.07)	0.391
Mulching	0.37 (0.48)	0.35 (0.48)	0.695	0.31 (0.46)	0.30 (0.46)	0.923	0.01 (0.08)	0.910
Strong fences	0.16 (0.37)	0.20 (0.40)	0.317	0.35 (0.48)	0.42 (0.49)	0.177	0.04 (0.07)	0.567
Vegetable species harvested:								
Whole year	9.19 (5.22)	9.31 (4.42)	0.831	10.78 (5.90)	12.33 (4.47)	0.049	1.42 (0.79)	0.073
Summer	3.66 (2.25)	3.52 (1.99)	0.587	3.28 (2.29)	3.84 (1.99)	0.033	0.70 (0.33)	0.037
Rainy	2.36 (2.00)	2.40 (1.69)	0.859	3.46 (1.97)	3.90 (1.57)	0.064	0.42 (0.25)	0.101
Winter	3.16 (2.23)	3.39 (2.11)	0.432	4.04 (2.35)	4.58 (1.89)	0.117	0.31 (0.36)	0.395

Notes: C=Control; T = Treatment. The numbers in parentheses indicate standard deviations for the means and standard errors for the average treatment effect (ATE). Summer (roughly from March to May), rainy season (roughly from June to September), and winter season (roughly from October to February).

**Table 4:** Baseline and endline means and average treatment effects for food practices as reported by caregivers.

Outcome (proportion of total)	Baseline			Endline			Impact	
	C	T	p-value	C	T	p-value	ATE	p-value
Include vegetables in meals	0.94 (0.14)	0.92 (0.16)	0.557	0.94 (0.15)	0.95 (0.14)	0.759	0.02 (0.03)	0.515
Children buying junk food	0.68 (0.25)	0.69 (0.25)	0.585	0.75 (0.22)	0.68 (0.24)	0.003	−0.08 (0.03)	<0.001
Children eat before school	0.97 (0.10)	0.97 (0.12)	0.861	0.97 (0.13)	0.99 (0.06)	0.106	0.02 (0.01)	0.103
Encourage children to eat vegetables	0.87 (0.20)	0.84 (0.23)	0.143	0.88 (0.18)	0.92 (0.16)	0.048	0.07 (0.03)	0.012
Eat dinner together	0.97 (0.11)	0.97 (0.12)	0.644	0.98 (0.08)	0.98 (0.09)	0.706	−0.01 (0.01)	0.577
Provide milk to children	0.65 (0.33)	0.65 (0.32)	0.855	0.69 (0.28)	0.68 (0.27)	0.575	−0.02 (0.03)	0.485
Cook meat for children	0.76 (0.22)	0.75 (0.21)	0.534	0.77 (0.21)	0.78 (0.21)	0.570	0.03 (0.03)	0.264
Children wash hands before eating	0.92 (0.17)	0.90 (0.20)	0.202	0.90 (0.17)	0.95 (0.14)	0.008	0.07 (0.03)	0.008
Average	0.77 (0.10)	0.76 (0.11)	0.331	0.77 (0.09)	0.80 (0.09)	0.008	0.04 (0.01)	<0.001

Notes: C=Control; T = Treatment. The numbers in parentheses indicate standard deviations for the means and standard errors for the average treatment effect (ATE).

The results in Table 2 show a positive effect of the intervention on the food and nutritional knowledge of caregivers (p < 0.001). The effect size is 0.14 percentage-points, which is a 26.4% increase over mean baseline levels. There was no effect on the food and nutritional knowledge children (p = 0.666) as the food and nutrition knowledge of the control and the treatment increased in parallel. This suggests that the nutrition education included in the school garden program did not add to the existing curriculum.

In terms of agricultural knowledge, children were able to correctly tell if a photo of an insect was that of an insect pest or of a beneficial insect for 52% of the photos shown on average. For caregivers this value was 59%. Considering that the questions were binary, the answers were only a little better than blind guesses. It thus indicates poor knowledge about insect pests and beneficial insects. The results show that the intervention had a small effect on the agricultural knowledge of the caregivers (+5.1% over baseline levels; p = 0.022), but not of the children (p = 0.119).

The results show that caregivers had a slightly stronger liking for vegetables than children, but the difference was small (about 4.5 percentage-points at baseline). There is a slightly wider gap between children's liking for vegetables and their caregivers' perception of children's liking (8.5 percentage-points), which suggests that children like vegetables more than their parents think they do. In terms of impact, the endline shows a stronger liking for vegetables in the treatment group than in the control group and the average increase is 6.1% for children (p = 0.070) and 10.2% for caregivers (p < 0.001). However, it is noted that the effect is not because average liking increased in the treatment group, but because average liking decreased in the control group. Caregivers' perception of how much their children like vegetables increased 10.5% (p < 0.001).

In the baseline, for 62% of the choice questions children stated to prefer healthier snacks over less healthy ones. There is an increase in the preferences for healthier snacks between baseline and endline with the treatment group showing a stronger increase. Overall, the ATE shows a 5 percentage-point increase in children's preferences for healthier snacks (p = 0.042), which is equivalent to an 8.1% improvement over baseline levels.

Turning to caregivers’ home gardens, we found the treatment group adopted practices such as own seed saving, pruning and removal of sick plants, but there was no effect on any of the other practices trained – though some were already used widely at the baseline (Table 3). The intervention had a positive effect on the number of different vegetables harvested from the home garden during the summer season (p = 0.037) while the effect during the rainy season was weaker (p = 0.101) and there was no effect during the winter season (the main season for leafy vegetables and brassicas) (p = 0.395). Altogether for the whole year, the treatment group increased the number of vegetables harvested by 1.4 species, which is a 15.4% increase over baseline levels (p = 0.073).

Caregivers reported improvements in terms of household food practices, including children buying less junk food (−11.7%), children eating before school (+2.1%), greater encouragement for children to eat vegetables (+8.2%), and children washing hands before eating (+7.7%) as shown in Table 4. There was no effect on the consumption of milk or meat, which is perhaps not surprising because these were not part of the home garden intervention, but also no increase in the inclusion of vegetables in meals, which was already high at the baseline and therefore had little room for improvement. The overall effect of the intervention on the adoption of healthy food practices was +5.2% over baseline conditions (p < 0.001).

In the final part of the analysis, we address our main hypothesis regarding children's food choices. The proportion of meals that included vegetables was calculated from children's food logbook data. The results were averaged by quarter, because there were missing observations for some months as a result of school breaks or illness (Table 5). The results show no effect for the first quarter of the year-long study period (p = 0.620), which is the period before the intervention got started in September. This result is important because it supports the parallel paths assumption discussed above. The ATE turns positive (p = 0.084 for Q2, p = 0.017 for Q3, p = 0.088 for Q4) for the subsequent three quarters. The effect sizes appear small, but represent a 15.1% increase over baseline levels for Q2, a 25.9% increase for Q3, and a 25.5% increase for Q4, which is substantial. It does therefore show that the intervention increased the frequency of vegetable consumption in the sample of school children. We note that the effect on production had a high p-value for the period from March to May (Table 3), while the effect on consumption has a high p-value for the period from January to March, which only partly overlaps, while there was a weak effect for the period from April to June (p = 0.088).

**Table 5:** Average treatment effect on the proportion of meals eaten that included at least one vegetable.

Period	Control mean (SD)	Treatment mean (SD)	p-value	Impact (ATE)	p-value	% change over baseline
Baseline (June) *	0.32	0.29	0.235			
	(0.22)	(0.21)				
Jul–Sep (Q1) *	0.26	0.24	0.387	0.01	0.620	+4.0
	(0.15)	(0.14)		(0.03)		
Oct–Dec (Q2)	0.26	0.27	0.545	0.04	0.084	+15.1
	(0.14)	(0.14)		(0.02)		
Jan–Mar (Q3)	0.25	0.29	0.053	0.07	0.017	+25.9
	(0.12)	(0.15)		(0.03)		
Apr–Jun (Q4)	0.22	0.25	0.212	0.06	0.088	+25.5
	(0.14)	(0.14)		(0.03)		

Notes: * Refers to the outcome indicator before the intervention was implemented. The numbers in parentheses indicate standard deviations for the means and standard errors for the average treatment effect (ATE). The ATE is relative to the baseline.

## 4 Discussion

### 4.1 Implications of the results

This study demonstrates that the combined school and home garden intervention improved children's liking for vegetables, their food practices, and the proportion of meals that included vegetables. We posit that this increase in vegetable consumption resulted from concurrently targeting children and caregivers and from the enhanced the availability of vegetables in children's homes. We found an increase in caregivers' knowledge of food and nutrition and in their knowledge of agriculture, and also an increase in the number vegetable species harvested from the home garden. This suggests that a more conducive food environment was created that enabled children to turn knowledge into healthy food practices. Changes in food practices at home—including caregivers encouraging their children to eat vegetables, increased handwashing, and reduced junk food consumption—are evidence of this.

Unfortunately, the study could not disentangle the separate contributions of the school and home garden interventions and the synergies between them. Doing this would have required a trial with four treatment arms. However, Schreinemachers et al. (2017a) evaluated the impact of a school garden intervention that did not include a home garden component and did not find a significant effect on vegetable consumption. This comparison therefore suggests that school gardens alone could not have created the positive effect on children's vegetable consumption. Still, we cannot rule out if the increase in children's vegetable consumption could have been achieved by a stand-alone home garden intervention. To our knowledge there are no studies of home garden interventions that have quantified the impact on children's vegetable consumption. Benkowitz et al. (2019) found a positive association between children having experience in growing vegetables at home and their intake of vegetables in a small and non-representative sample of German school children. Further studies will be needed to analyze the effect of home gardens on children's vegetable intake.

Our results show that school garden interventions need to be designed in such way that they do not only stimulate children's knowledge of and preferences for vegetables, but also increase children's access to vegetables at home as well as stimulate parents to prepare and eat more vegetables. This finding supports the Blanchette and Brug (2006) who concluded that multi-component school-based interventions have the greatest promise for increased fruit and vegetable promotion among children. It also supports Rasmussen et al. (2006) and Scaglioni et al. (2018) who showed that children will eat more fruit and vegetables if they have better access to fruit and vegetables at home and if their parents also eat more of them.

The need for comprehensive intervention designs is increasingly recognized in the agriculture-nutrition literature. Several authors have pointed at the need for multi-sectoral programming, combining or aligning agriculture, nutrition, education and health interventions to optimize impact (Burchi et al., 2011; Cunningham et al., 2017; McDermott et al., 2013). Nepal's multi-sectoral nutrition strategy likewise emphasizes the value of such multi-sectoral approach (Government of Nepal, 2017). An integrated home and school garden program fits to such strategy.

### 4.2 Strengths and weaknesses of the study

Given the lack of rigorous studies, as discussed above, we make a significant contribution to strengthening the quality of the existing evidence. Another contribution is that the study measured a range of outcome variables along the intervention's impact pathway from knowledge and preferences to changes in food behavior. For instance, the positive effect on the proportion of meals that included vegetables was supported by a positive effect on the number of species harvested from home gardens.

We originally planned to use the individual dietary diversity score as an outcome variable, but baseline data showed that most children already consumed vegetables on a daily basis and the measure was therefore not sensitive enough to pick up improvements in the quantity or frequency of vegetable consumption. Estimating quantities of vegetables consumed does not seem realistic to accomplish for children aged 8–12 years. Depending on study site circumstances, future studies in this area are therefore advised to use food frequency measures and not rely on dietary diversity scores.

The present study has certain limitations. First, this was a two-year research project and we therefore could only study the immediate, one-year effect of the intervention. It would have been valuable to do a longer-term study as behavior change is generally understood as a slow process and the small effect sizes found for some of the impact indicators may become larger (or smaller) over time. It is also important to study the intervention's sustainability. Second, self-reported data in non-blinded trials have a risk of social desirability bias (van de Mortel, 2008). We think that this risk is low in our data. Enumerators observed that children gave honest answers and were not trying to give “correct” answers. Still, we could have explored this type of potential bias more. Third, we were only able to include two treatment arms in the trial while it would have required four treatment arms to disentangle the separate and combined effects of the home and school garden interventions.

## 5 Conclusion

A school garden intervention in Nepal was coupled to a complementary home garden intervention targeting children's caregivers and aimed at increasing household availability of vegetables and at promoting caregivers' preferences for vegetables. This study showed that such comprehensive intervention design was able to increase children's vegetable consumption by 15–26%, measured in terms the proportion of meals that included vegetables. These results point at the importance of comprehensive intervention designs (as opposed to school gardens as a standalone intervention) that aim to affect food behavior not just at the individual level, but at the household and community levels. The policy implication is that school gardens in low-income countries must not only try to influence children's food preferences and food behavior but it is important that they also address the availability of nutritious food in households and the caregivers' corresponding preferences and behavior.

## Declaration of competing interest

The authors declare that they have no known competing financial interests or personal relationships that could have appeared to influence the work reported in this paper.
